# 

**Published:** 1900-07-21

**Authors:** 


### NOTICE TO CORRESPONDENTS.

All MS., Letters, Books for Review, and other matters intended for the Editor should be addressed The Editor, "The Hospital," The Hospital Building, 28 & 29, Southampton Street, Strand, W.C. The Editor cannot undertake to return rejected MS., even when accompanied by stamped directed envelope.

**Notice to Advertisers and Subscribers.**

All Advertisements, Orders for Copies of The Hospital, and Business Communications should be addressed to **THE MANAGER (Not to the Editor),** The Hospital, The Hospital Building, 28 & 29, Southampton Street, Strand, London, W.C.

The Cost of Subscription, post free, is as follows:—Per week, 2½d.; per quarter, 2s. 8d.; per half-year, 5s. 3d.; per annum, 10s. 6d. Foreign:—Per annum, 15s. 2d., payable, in all cases, in advance.

**NOTICE.—Vol. XXVII. of "The Hospital" (October, 1899, to April, 1900), in handsome cloth binding, is now on sale at the Office of this Journal, price 7s. 6d. Cases for binding the Numbers contained in this Volume 1s. 6d. Index to Vol. XXVII., 2½d., post free. The Monthly Part for August will be ready on August 1st. Price 6d., by post 8d.**

### BOOKS RECEIVED.

Charles Griffin and Co.

"Year-Book of the Scientific and Learned Societies."

Charles William Deacon and Co.

"Before Good-Night" and "From Door to Door." By George H. R. Dabbs, M.D.

S. and W. J. King, Ipswich.

"Annual Report on the Borough and Port of Ipswich." By George Sampson Elliston, Medical Officer of Health."

J. W. Ward, Croydon.

"Where Should Londoners Live?"

Simpkin, Marshall, Hamilton, Kent, and Co.

"The Failure of Surgery in Cancer." By Samuel Kennedy, L.R.C.P. and S.

Her Majesty's Stationery Office.

"Annual Report of the Army Veterinary Department."

Marshall Brothers.

"Carest Thou Not that we Perish? The Bitter Cry of Starving India." By J. T. Budd.

Jarrold and Sons.

"Weekly Day and Night Chart." Designed by Cecil A. P. Osburne, F.R.C.S.E.

John Wright and Co.

"Golden Rules of Ophthalmic Practice." By Gustavus Hartridge, F.R.C.S.

Kelly's Directories.

"Kelly's Directory of Chemists and Druggists," 1900.

West, Newman, and Co.

"Archives of Surgery." By Jonathan Hutchinson, LL.D., F.R.S.

### THE NURSE'S REPORT BOOK.

Foolscap 4to., Black cover, **6d.** net. By Post 8d.

**Compiled by K. H. (Cert.)**

H. J. Glaisher, 57, Wigmore Street, Cavendish Square, W.

ITALIAN LAKES, SWITZERLAND, TYROL.—Dr. and Mrs. HARDWICKE'S PERSONALLY-CONDUCTED TOURS, via Calais, Basle, August 29. Hotels Included. Venice, Florence, Paris Extensions.

13, MAGDALEN TERRACE, ST. LEONARDS.

